# Downregulation of *TRMT9B* and its association with clinicopathological features in thyroid cancer: an analysis of public datasets

**DOI:** 10.31744/einstein_journal/2026AO2165

**Published:** 2026-10-13

**Authors:** Isadora Biazon de Souza, Larissa Mikalauskas, Felipe Yuji Koyama Azeka, Alexander de Moraes Frankiw, Nathan Mignella Greco, Maria Alice Kawashita Dalla Vecchia, Helena Panteliou Lima-Valassi, Alessandra Valéria de Sousa Faria

**Affiliations:** 1 Hospital Israelita Albert Einstein Faculdade Israelita de Ciências da Saúde Albert Einstein São Paulo SP Brazil Faculdade Israelita de Ciências da Saúde Albert Einstein, Hospital Israelita Albert Einstein, São Paulo, SP, Brazil.; 2 Universidade de São Paulo Faculdade de Medicina São Paulo SP Brazil Laboratório de Hormônios e Genética Molecular LIM/42, Faculdade de Medicina, Universidade de São Paulo, São Paulo, SP, Brazil.; 3 Institute of Biomedical Sciences Department of Cell and Developmental Biology São Paulo SP Brazil Institute of Biomedical Sciences, Department of Cell and Developmental Biology, Universidade de São Paulo, São Paulo, SP, Brazil.

**Keywords:** TRMT9B, Thyroid neoplasms, tRNA methyltransferases, Genes, tumor suppressor

## Abstract

**Objective:**

To investigate the association between *TRMT9B* expression and clinicopathological features in thyroid carcinoma.

**Methods:**

*TRMT9B* mRNA expression was analyzed using The Cancer Genome Atlas Thyroid Cancer (TCGA-THCA) and Genotype-Tissue Expression (GTEx) datasets as well as six independent Gene Expression Omnibus (GEO) datasets. Protein-level data were obtained from The Human Protein Atlas. Associations between *TRMT9B* expression and clinicopathological parameters were evaluated in the TCGA cohort.

**Results:**

*TRMT9B* mRNA was significantly downregulated in thyroid tumors compared with normal tissues across multiple cohorts. In the TCGA cohort, lower *TRMT9B* expression was significantly correlated with advanced T stage, lymph node involvement, and aggressive histological variants of papillary thyroid carcinoma. Immunohistochemistry data qualitatively supported these findings: protein staining was lower in tumor samples than in healthy tissue.

**Conclusion:**

*TRMT9B* is downregulated in thyroid carcinoma and is associated with markers of disease progression. These findings suggest that *TRMT9B* plays a role in thyroid tumor biology. Further functional studies are required to clarify its mechanisms and potential clinical utility as a biomarker.

## Highlights

■ *TRMT9B* is downregulated in thyroid carcinoma.
■ The columnar cell histological subtype exhibits lower *TRMT9B* expression.
■ Younger patients (<55 years old) have lower *TRMT9B* expression.

## In Brief

*TRMT9B* is downregulated in thyroid carcinoma, especially in the more aggressive columnar cell histological subtype and, within the follicular variant, in younger patients (<55 years old), implying a role in thyroid tumor biology. Future studies should clarify its molecular contribution to cancer progression.

## Graphical abstract

**Figure:**
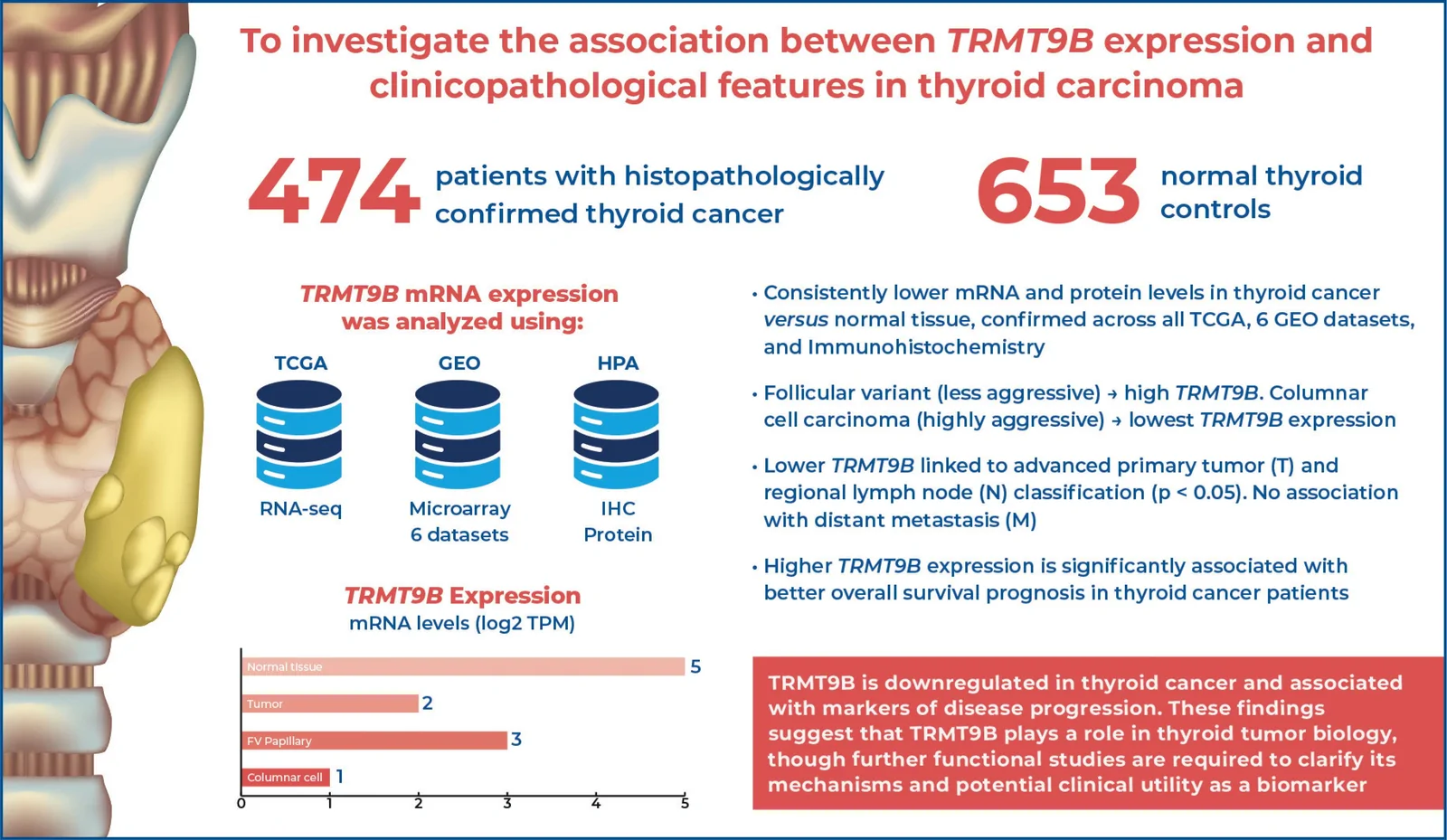

## INTRODUCTION

Transfer RNAs (tRNAs) are the adapter molecules of protein synthesis that ensure precise pairing between amino acids and mRNA codons during translation. This function is especially critical at split codon boxes, where accurate wobble base recognition is required to maintain translational fidelity.^(1)^ In addition to their primary structure, tRNAs undergo extensive posttranscriptional modifications, most notably methylation, which are indispensable for proper folding, stability, and functional integrity.^(2,3)^ tRNA methyltransferase 9-like (*TRMT9B*), also known as *TRMT9L*, C8orf79, or *KIAA1456*, is one such enzyme governing these modifications. It catalyzes the methylation of wobble bases in specific tRNAs, particularly those decoding arginine and glutamine.^(4–6)^ Loss of TRMT9B activity impairs tRNA performance and proteostasis, triggering major cellular stress pathways mediated by Rb and p53.^(6)^

TRMT9B serves as a tumor suppressor in various malignancies, where its expression is inversely correlated with tumor aggressiveness. In ovarian cancer, for instance, lentiviral-mediated TRMT9B overexpression triggers proapoptotic signaling through induction of Bax and suppression of the antiapoptotic protein Bcl-2.^(6)^ In colorectal cancer, TRMT9B induces a senescence-like phenotype by causing G0/G1 cell cycle arrest and disrupting the critical association between HIF1-*α* and GLUT1 expression, thereby impairing the metabolic adaptation of tumor cells to hypoxic environments.^(5,7)^ In lung cancer, robust TRMT9B expression serves as a favorable prognostic indicator, being correlated with improved 5-year overall survival rates after surgery. At the molecular level, TRMT9B blocks the G1/S transition by inhibiting cyclin D1 expression, thereby halting cellular proliferation.^(8)^ Furthermore, it potently inhibits the metastatic cascade by preventing the epithelial-mesenchymal transition through E-cadherin upregulation and reciprocal N-cadherin downregulation, ultimately reducing the migratory and invasive potential of these cells.^(9)^ Furthermore, TRMT9B is sensitive to the tumor microenvironment; oxidative stress can lead to its inhibitory phosphorylation via the Erk/MAPK pathway, promoting reactive oxygen species accumulation and genomic instability.^(5–9)^ Nevertheless, TRMT9B's involvement in thyroid carcinogenesis remains unexplored.

Thyroid cancer is the most prevalent endocrine neoplasm, with papillary thyroid carcinoma (PTC) representing 80%-85% of cases.^(10–16)^ Although PTC generally presents an indolent course and excellent prognosis, its increasing incidence and the existence of aggressive subtypes, such as anaplastic carcinoma, necessitate the identification of novel molecular markers.^(10–19)^ Given that thyroid cancer subtypes arise from distinct cell types (follicular *versus* parafollicular cells), their regulatory landscapes, including tRNA modification pathways, may differ significantly.^(17)^ Investigating the role of *TRMT9B* in thyroid cancer may help uncover novel mechanisms of tumor progression and identify potential therapeutic targets.

## OBJECTIVE

In this study, we evaluated *TRMT9B* expression in thyroid cancer samples and analyzed its association with clinicopathological features and histological subtypes.

## METHODS

### Public repository data and access

This study integrated high-throughput genomic, transcriptomic, and proteomic data from four public repositories: the Cancer Genome Atlas Thyroid Cancer (TCGA-THCA) data collection,^(19)^ the Genotype-Tissue Expression (GTEx) project, the Gene Expression Omnibus (GEO), and the Human Protein Atlas (HPA).^(21–23)^ We evaluated *TRMT9B* mRNA and protein expression patterns in thyroid cancer compared with normal thyroid tissue, followed by an association analysis with clinicopathological parameters.

### Compliance with ethical standards

The data used in this article are available in public repositories, thus obviating the necessity for ethical approval and informed consent. Ethics oversight and written informed consent had been obtained through the TCGA project.

### *TRMT9B* mRNA expression in thyroid cancer using TCGA data

#### Transcriptomic data

*TRMT9B* mRNA expression data (transcripts per million [TPM]) were retrieved from the TCGA-THCA cohort using the TCGAbiolinks package^(20)^ in R software (v4.4.2).^(21)^ To supplement the control group, normal thyroid tissue data were obtained from the Genotype-Tissue Expression (GTEx) project via the HPA portal.^(21–23)^ A summary of public data repositories and their characteristics is provided in table 1.

**Table 1:** Summary of public data repositories and information flow

Public data repository	Data type acquired	Patient information acquired	Purpose in study
The Cancer Genome Atlas (TCGA) (NIH Resource)	RNA sequencing (RNA-Seq): TRMT9B expression (TPM values)	Clinicodemographic data (age, sex, subtype, stage)	Primary analysis of differential expression, clinical association, and subgroup analysis
The Genotype-Tissue Expression (GTEx) project (NIH Resource)	RNA sequencing (RNA-Seq): normal thyroid tissue expression data (TPM values)	Demographic data (age and sex)	Source of normal thyroid samples, acknowledging potential intercohort variability
Gene Expression Omnibus (GEO) (NIH Resource)	Microarray data: TRMT9B expression (6 independent studies)	Study details (patient count, normal *versus* tumor)	External validation of the primary finding (Tumor *versus* Normal)
The Human Protein Atlas	Immunohistochemistry (IHC) images: TRMT9B protein staining intensity	Publicly available patient and sample information for IHC slides	Corroboration of mRNA findings at the protein level

### Patient characteristics and study population

The final study population comprised 474 patients with histopathologically confirmed thyroid cancer and 653 normal thyroid controls (Figure 1). To enhance statistical power, histological nomenclature for papillary thyroid adenocarcinoma and papillary thyroid carcinoma were combined into a single papillary thyroid carcinoma (PTC) group.^(24)^ Clinical staging was standardized according to the AJCC TNM staging system (8th edition), which incorporates tumor size (T), nodal involvement (N), distant metastasis (M), and a 55-year age cutoff^(25)^ (Table 2).

**Figure 1:**
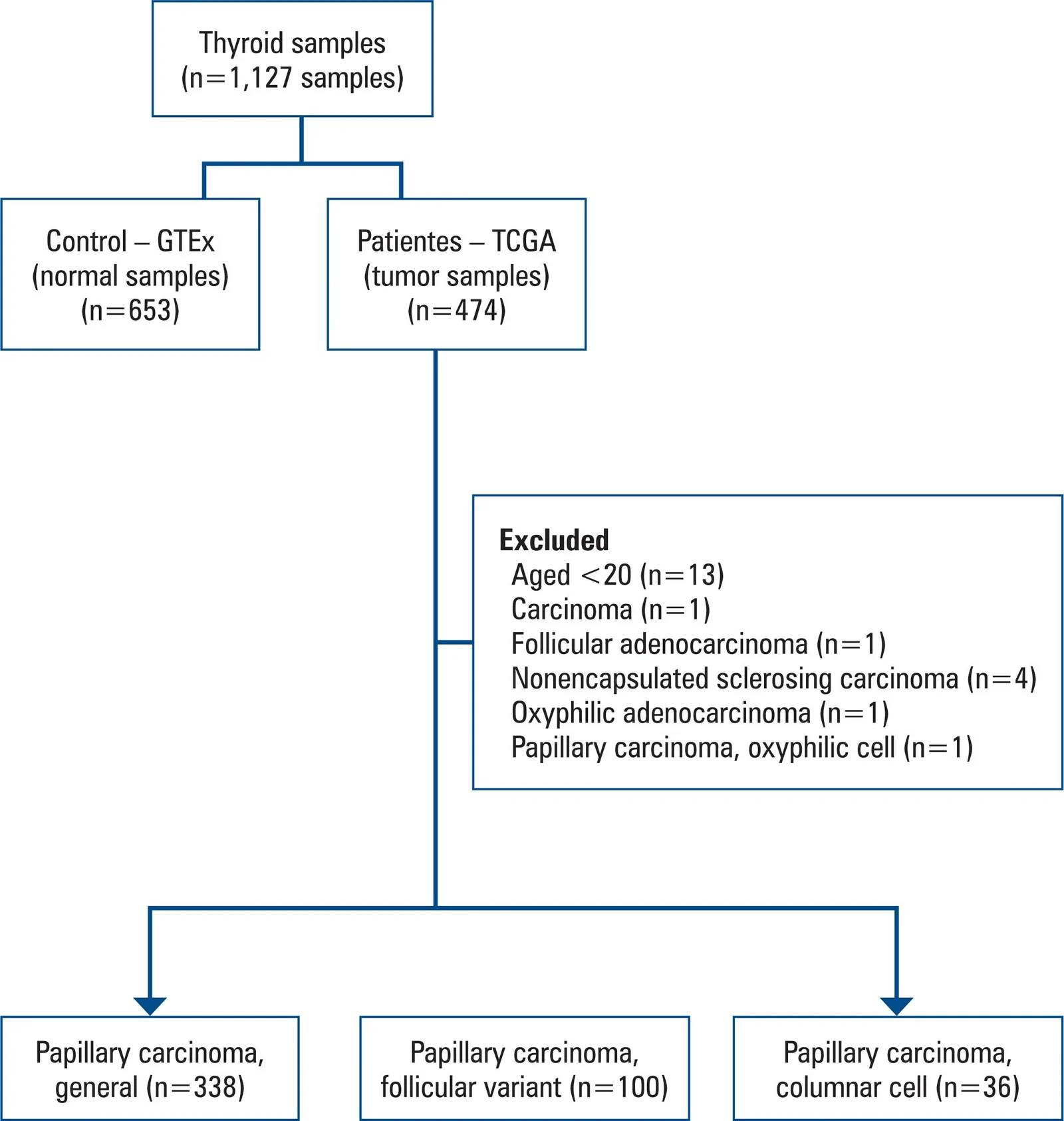
Flowchart summarizes the samples used in the study, highlighting the exclusion of patients younger than 20 years old, and histological subtypes of thyroid cancer with n<10

**Table 2:** Demographic characteristics of the study cohort

Characteristics		Patient group (n=474)	Control group (n=653)	p value* (χ^2^ test)
Gender, n (%)				<0.001
	Male	126 (26.5)	434 (66.5)	
	Female	348 (73.5)	219 (33.5)	
Age Group (years), n (%)				<0.001
	20-29	51 (10.7)	47 (7.0)	
	30-39	113 (23.8)	51 (8.0)	
	40-49	94 (19.8)	110 (17.0)	
	50-59	100 (21.3)	211 (32.0)	
	60-69	66 (13.9)	212 (32.0)	
	70+	50 (10.5)	22 (3.0)	

* Pearson's χ^2^ test.

### Inclusion and exclusion criteria

Inclusion required high-quality RNA-seq data and complete demographic information (age and sex). We excluded patients who were younger than 20 years; had histological subtypes with <10 representatives (e.g., follicular adenocarcinoma, nonencapsulated sclerosing carcinoma, and oxyphilic cell variants) to avoid statistical bias; or had samples with significant tumor impurity or ambiguous pathological staging.

### Immunohistochemistry (The Human Protein Atlas)

To corroborate the mRNA findings, TRMT9B protein expression was qualitatively assessed using immunohistochemistry images from the HPA.^(26)^ Staining intensity (low, medium, or high) was evaluated in four thyroid cancer samples and two normal thyroid controls. Because of the limited sample size, these data were used only for descriptive and hypothesis-generating purposes.

### External validation of *TRMT9B* expression in GEO datasets

A systematic search in the GEO database using the MeSH term "thyroid cancer" (filtered for *Homo sapiens* and "expression profiling by array") initially identified 98 studies. Six independent datasets (Table 3) were selected based on (a) inclusion of both PTC and normal tissue, (b) availability of raw or normalized data, and (c) sufficient sample size for differential expression analysis. These datasets, encompassing various platforms (Affymetrix, Agilent, and Illumina), were analyzed using the GEO2R tool.^(27–29)^ Each study was analyzed independently to mitigate interplatform batch effects.

**Table 3:** Summary of papillary thyroid carcinoma gene expression studies from the GEO datasets

Study	Reference and Platform	Brief description of cohort	Number of normal samples	Number of tumor samples
GSE6004	Affymetrix^(30)^ Human Genome U133 Plus 2.0 Array	Investigates gene expression differences between central and invasive regions of PTC, highlighting epithelial-to-mesenchymal transition mechanisms associated with tumor invasion	16	28
GSE3678	Affymetrix^(31)^ Human Genome U133 Plus 2.0 Array	Compares gene expression between PTC and matched normal thyroid tissue to identify potential molecular markers	28	28
GSE299988	Agilent-072363^(32)^ SurePrint G3 Human GE v3 8x60K Microarray	Evaluates gene expression differences related to lymph node metastasis and radioiodine refractoriness in PTC	4	10
GSE104006	Illumina^(33)^ HumanHT-12 WG-DASL V4.0 R2	Profiles gene and miRNA expression across thyroid cancer histotypes	5	19
GSE50901	Agilent-028004^(34)^ SurePrint G3 Human GE 8x60K Microarray	Identifies deregulated pathways and diagnostic markers in PTC using reference-based hybridization	4	61
GSE27155	Affymetrix^(35)^ Human Genome U133A Array	Comprehensive dataset including multiple thyroid tumor subtypes and mutation profiles (BRAF, RAS, RET/PTC, PAX8/PPARG)	40	87

### Statistical significance and software used

mRNA expression values (TPM) were log2-transformed to normalize distribution and stabilize variance, as confirmed with Q-Q plots. Differential expression was assessed using the Student *t* test or Welch *t* test (for two-group comparisons); one-way or two-way analysis of variance (ANOVA; followed by Welch ANOVA where variances were unequal) for multiple-group comparisons and clinicopathological associations; and the chi-square test for demographic distributions between cohorts. Statistical significance was defined as *α*<0.05. All analyses were performed using R (version 4.4.2) and jamovi (version 2.6).

## RESULTS

### Thyroid cancer occurs predominantly in women

The study analyzed 1,127 individuals, including 474 patients with thyroid cancer and 653 normal thyroid controls. Significant between-group demographic disparities were noted (Table 2; p<0.001). The tumor cohort exhibited a female preponderance (73.5%), whereas the control group had more men (66.5%). Regarding age, controls were concentrated in the 50-69 range, whereas patients with cancer showed a broader distribution (30-59 years). Histological characterization identified PTC as the most prevalent subtype, representing 92.4% of the cohort (Figure 1). Most patients were diagnosed at early clinical stages (Stages I and II; 56%; Table 4), with 52% staged as M0 (Table 5) and 46% as N0 (Table 6). In terms of the size and local spread of the primary tumor, the distribution was predominantly between T1 and T3 (Table 7).

**Table 4:** Number of patients in the AJCC clinical stages

	Number of patients n (%)
I	214 (45)
II	50 (11)
IV A	7 (1)
IV B	3 (1)
Not informed	200 (42)

**Table 5:** Number of patients in the AJCC M classification

	Number of patients n (%)
M0	217 (52)
M1	6 (1)
Not informed	197 (47)

**Table 6:** Number of patients in the AJCC N classification

	Number of patients n (%)
N0	219 (46)
N1	208 (44)
Not informed	47 (10)

**Table 7:** Number of patients in AJCC T classification

	Number of patients n (%)
T1	138 (29)
T2	156 (33)
T3	158 (33)
T4	20 (4)
Not informed	2 (0.4)

### *TRMT9B* mRNA and protein downregulation in thyroid tumors

*TRMT9B* mRNA expression was significantly downregulated in thyroid tumor samples compared with normal tissue (mean 2.39±1.15 *versus* 4.92±0.59 TPM, respectively; p<0.0001; Figure 2A). This reduction remained consistent regardless of age (p=0.68; Figure 2B) or sex (p=0.964; Figure 2C), suggesting that the observed downregulation was a tumor-specific event rather than a demographic artifact. To validate these findings at the protein level, we performed a qualitative immunohistochemical analysis using HPA data. Normal thyroid tissues exhibited strong and diffuse cytoplasmic staining (Figure 3A-B). In contrast, thyroid carcinomas, particularly PTC samples, showed a heterogeneous and predominantly weak staining pattern (Figure 3C-D).

**Figure 2:**
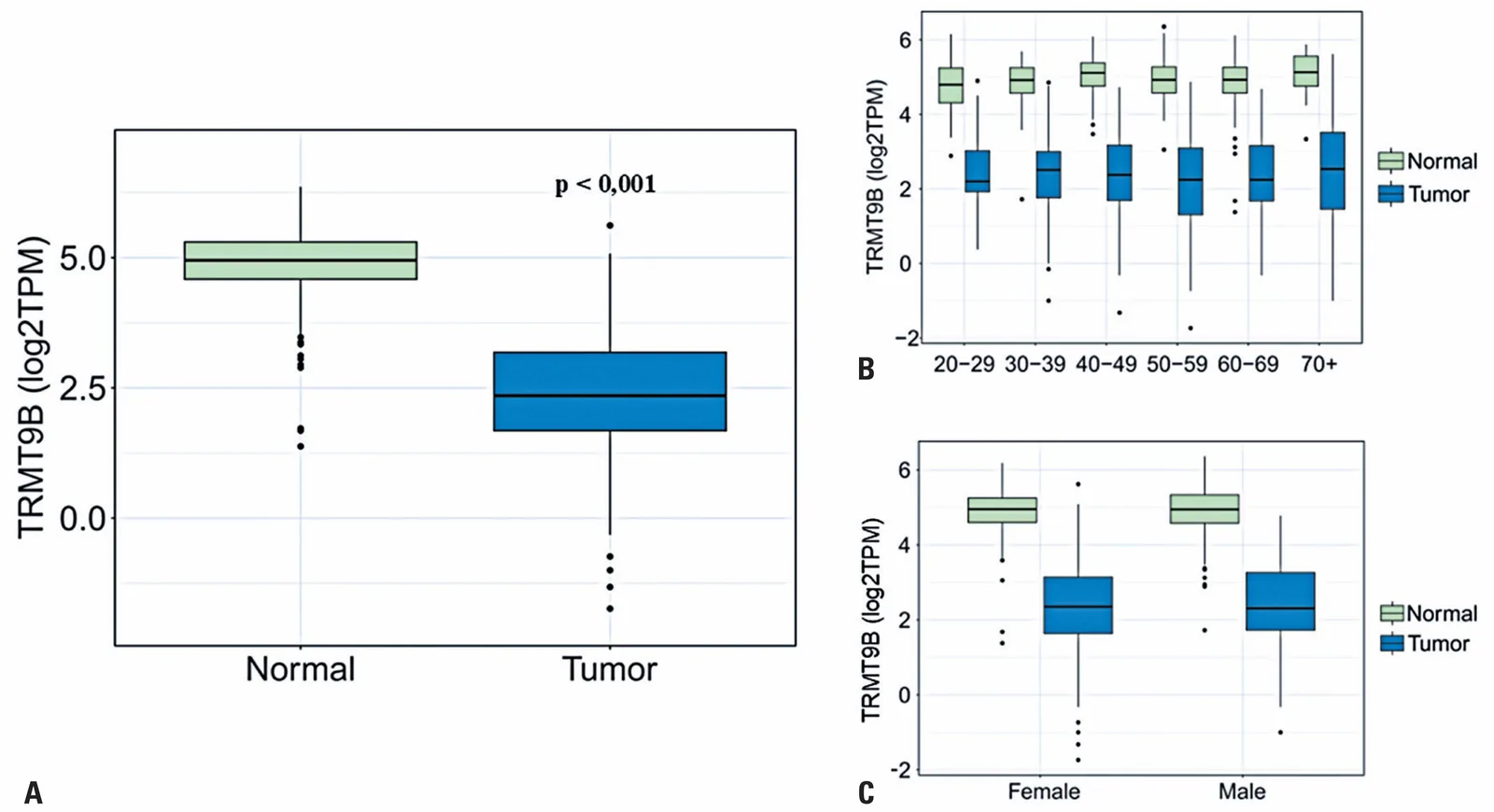
*TRMT9B* mRNA expression between normal and tumor samples. (A) The box plot shows that *TRMT9B* mRNA expression is significantly reduced in thyroid tumor samples compared with normal tissue (p<2e-16, the Student t test). (B) Box plot displaying the distribution of *TRMT9B* mRNA expression in normal and tumor samples stratified by age group (p=0.68, two-way ANOVA with unequal variances). (C) Box plot displaying the distribution of *TRMT9B* mRNA expression in tumor samples stratified by sex (p=0.964, two-way ANOVA with unequal variances)

**Figure 3:**
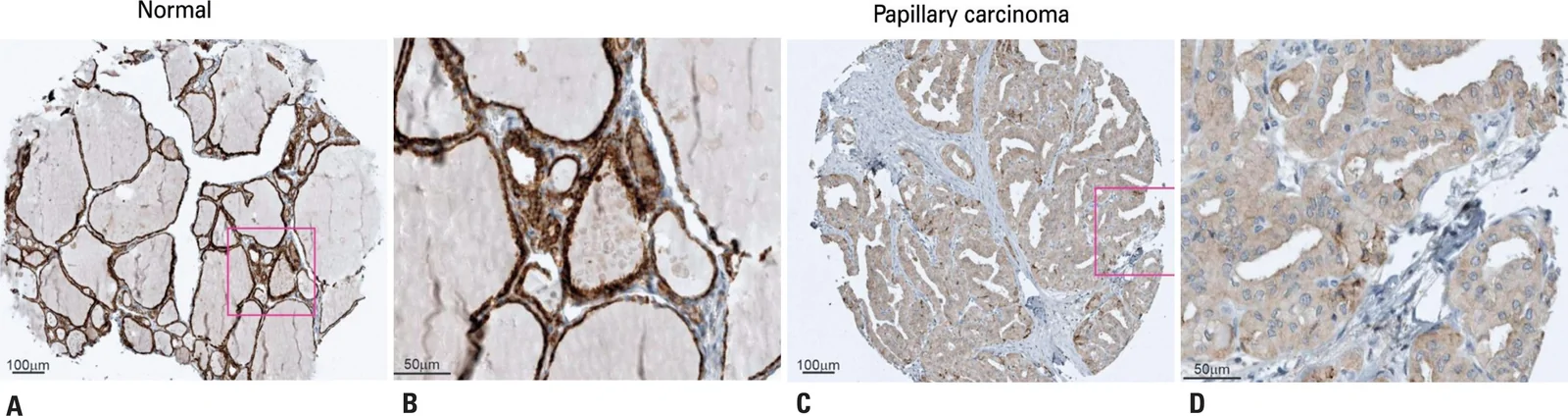
Immunohistochemical analysis of TRMT9B expression in thyroid tissues. The immunohistochemical staining patterns for TRMT9B protein expression revealed differences between normal and tumor tissues. (A-B) Normal tissue samples exhibited strong staining intensity. (C-D) Tumor samples, particularly papillary carcinomas, showed weaker staining intensity. The analysis was based cytoplasmic/membranous staining in normal thyroid (glandular cells) and papillary carcinoma (tumor cells) tissues. Scale bars: 100 μm (A, C) and 50 μm (B, D)^(21–23,26)^

### Multicohort validation (GEO Datasets)

External validation using six independent GEO datasets (97 normal *versus* 233 tumor samples) corroborated the TCGA findings. Across studies (GSE6004, GSE3678, GSE299988, GSE104006, GSE50901, and GSE27155), multiple *TRMT9B* transcripts showed a recurrent trend of higher expression in normal thyroid tissue compared with tumor samples. Although the magnitude of differential expression and specific p-values varied across individual transcripts and cohorts, a significant downregulation (p<0.05) was confirmed in at least one major transcript per dataset, reinforcing *TRMT9B* suppression in thyroid tumorigenesis (Figure 4).

**Figure 4:**
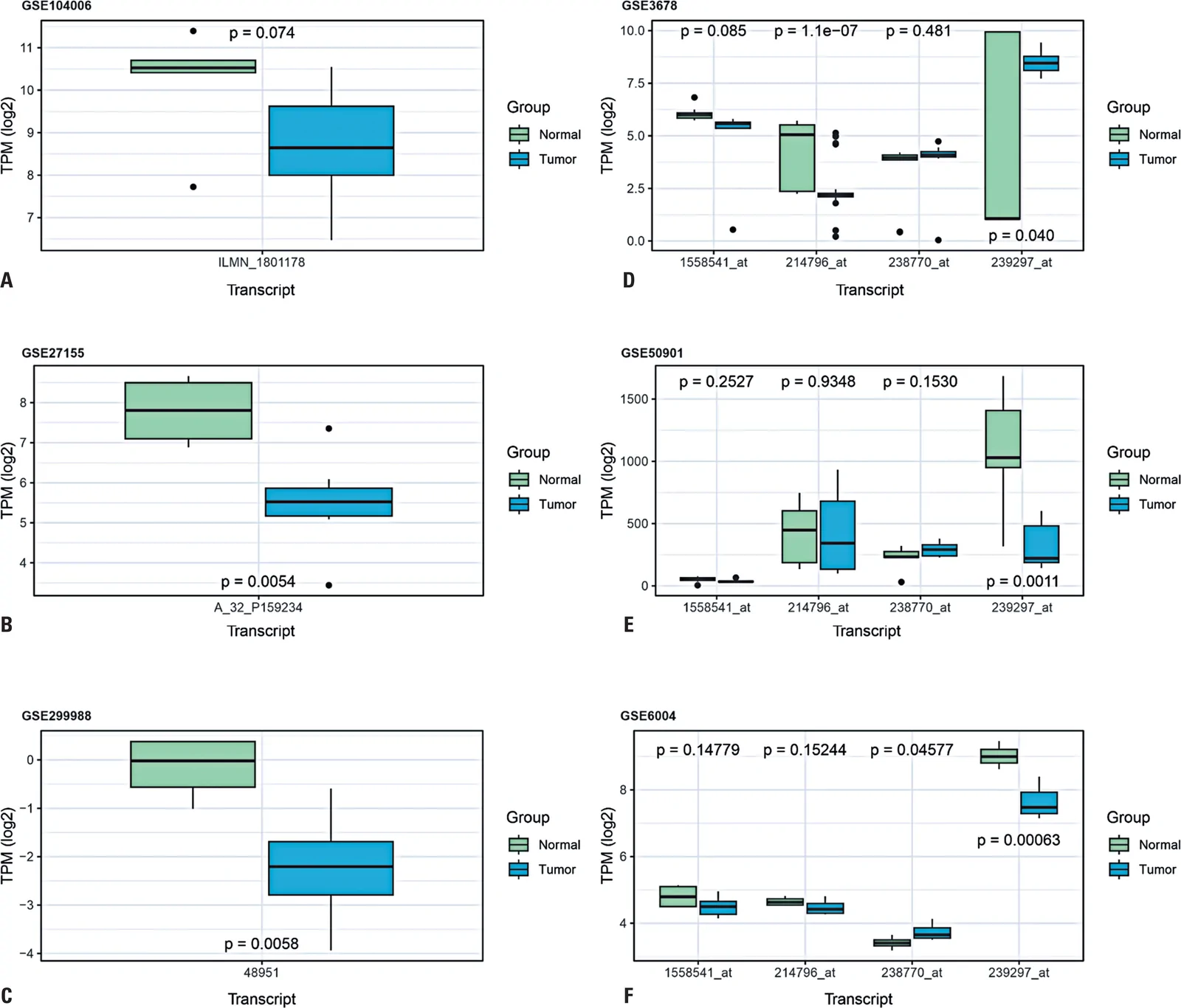
*TRMT9B* expression of thyroid carcinoma across multiple studies. Boxplots display the log2-transformed transcripts per million (TPM) of *TRMT9B* in normal (N) versus tumor (T) tissues from six GEO datasets. Each panel represents an independent cohort, with the GSE number and associated p value for the differential expression analysis shown above the plots. For studies with multiple transcripts of the same gene, each transcript is represented by a separate boxplot. Sample sizes (normal *versus* tumor) were as follows: GSE6004 (16 *versus* 28); GSE3678 (28 *versus* 28); GSE299988 (4 *versus* 10); GSE104006 (5 *versus* 19); GSE50901 (4 *versus* 61); and GSE27155 (40 *versus* 87). P values in panels D-F are derived from two-way ANOVA, whereas those from panels A-C are from the Student t test

### Association with PTC subtypes and clinical aggressiveness

We investigated *TRMT9B* expression across PTC histological variants and staging (Figure 5). Expression levels varied significantly by subtype (p<0.001): the follicular variant exhibited higher *TRMT9B* levels, whereas the more aggressive columnar cell variant exhibited the lowest expression (Figure 5A). Initial analysis using one-way ANOVA demonstrated significant associations between TRMT9B expression and both primary tumor size (T, p=0.006) and regional lymph node involvement (N, p<0.001) classifications in the overall cohort, and no significant association was observed with distant metastasis (M). However, these associations were no longer significant after stratification by histological subtype by using a two-way ANOVA (p=0.633 for T and p=0.106 for N; Figure 5B-C), indicating that the differences did not persist when the samples are divided by histological type. When stratifying by the AJCC 8th edition age cutoff (55 years), younger patients (aged <55 years) with the follicular variant exhibited significantly lower TRMT9B expression than older patients (aged >55 years) in the same histological group (mean 2.91 *versus* 3.56 TPM; p=0.0032).

**Figure 5:**
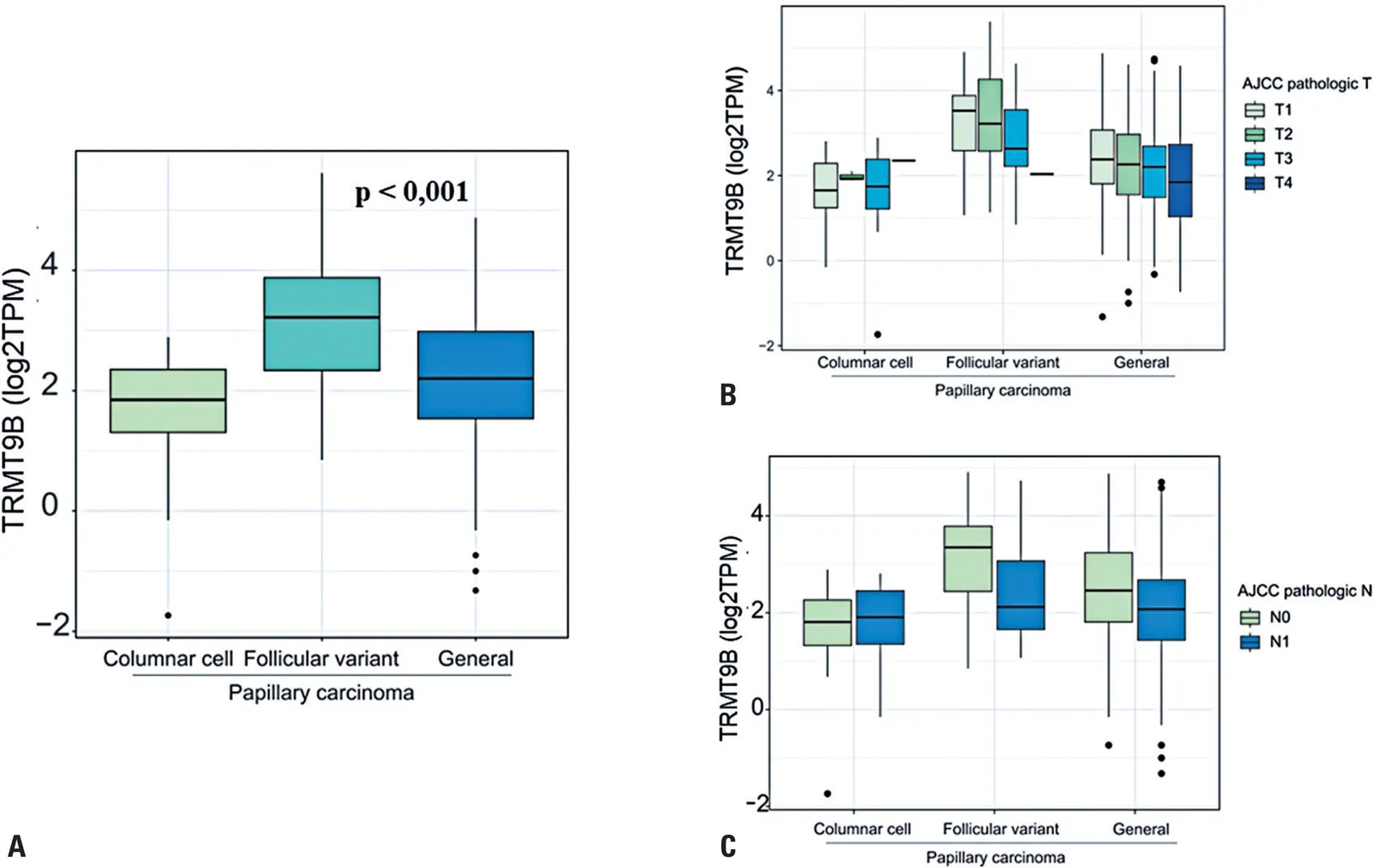
*TRMT9B* mRNA expression by papillary carcinoma subtypes and staging. Boxplots display the log-transformed *TRMT9B* mRNA expression (TPM) for the papillary carcinoma samples. (A) *TRMT9B* mRNA expression stratified by overall papillary carcinoma subtypes (p<0.001, one-way ANOVA). (B-C) *TRMT9B* mRNA expression across various AJCC classifications: (B) primary tumor (T classification) (p=0.633, two-way ANOVA), (C) regional lymph nodes (N classification) (p=0.106, two-way ANOVA). Boxplots are colored by subtype: columnar cell, follicular variant, and general. Boxes represent the interquartile range (IQR), with the horizontal line indicating the median

## DISCUSSION

Translation fidelity is fundamental to cellular homeostasis, and its disruption is increasingly recognized as a driver of proteotoxic stress and oncogenesis.^(1,36,37)^ In this study, we provide the first evidence that *TRMT9B*, a regulator of tRNA wobble base modification, is significantly downregulated in thyroid cancer. Derived from the large-scale TCGA-THCA cohort and validated across six independent GEO datasets, suggest that loss of *TRMT9B* expression is a recurrent event in thyroid tumorigenesis, aligning with its established role as a tumor suppressor in other malignancies.^(5,6)^

In colorectal, breast, and ovarian cancers, *TRMT9B* restrains tumor progression by promoting cell cycle arrest and activating p53-dependent apoptotic pathways.^(5–7)^ Our observation of reduced *TRMT9B* mRNA and protein levels in thyroid tumor tissues suggests that a similar suppressive mechanism may be at play. Mechanistically, the absence of *TRMT9B* leads to defective tRNA performance, resulting in the accumulation of reactive oxygen species and DNA damage.^(6,7)^ In the context of thyroid cancer, a disease characterized by diverse histological subtypes and varying degrees of aggressiveness, this translational instability may facilitate the acquisition of aggressive phenotypes.

A key finding was the inverse association between *TRMT9B* expression and clinical aggressiveness. We observed that the columnar cell variant, a rare but highly aggressive PTC subtype with high metastatic potential, exhibited significantly lower *TRMT9B* levels compared with the more indolent follicular variant.^(38–40)^ This pattern was further corroborated by the association of lower *TRMT9B* expression with advanced primary tumor (T) and regional lymph node (N) classifications. Notably, the lack of significant association with distant metastasis (M) suggests that *TRMT9B* modulation may be more critical during the early stages of local invasion and regional lymphatic spread than late-stage hematogenous dissemination.

Our data also revealed age-specific regulation within the follicular variant of PTC. Although thyroid cancer generally shows a female predominance and age-dependent prognosis,^(41–44)^ patients younger than 55 years exhibited lower *TRMT9B* levels than their older counterparts. This finding is particularly relevant given the AJCC 8th edition staging guidelines, which use the 55-year cutoff as a primary prognostic divider.^(38)^ The molecular basis for this age-dependent expression warrants further investigation, as it may reflect distinct epigenetic or hormonal regulatory landscapes in younger versus older patients.

Despite the strengths of this *in silico* validation, our study has limitations. First, the cross-platform microarray data introduce technical variability. The datasets used herein were generated using Affymetrix, Agilent, and Illumina platforms, which differ in probe design and sensitivity. This likely explains why specific probes (e.g., 239297) showed more consistent statistical significance than others. Nevertheless, the overall trend across 97 normal and 233 tumor samples remains robust, reinforcing the biological relevance of *TRMT9B* downregulation. Second, immunohistochemical analysis is qualitative in nature. Third, the study relied entirely on public datasets. Future prospective studies should examine the functional crosstalk between *TRMT9B* and thyroid-specific signaling pathways, such as the MAPK and PI3K/Akt axes, to elucidate how tRNA modification influences stress responses and therapeutic resistance in thyroid carcinoma.

## CONCLUSION

Our findings identify TRMT9B as a significant player in thyroid tumor biology. The downregulation of TRMT9B mRNA and protein in malignant thyroid tissues, particularly in aggressive histological subtypes and advanced pathological stages, supports its potential as a valuable biomarker. These associations also highlight the tRNA methylation pathway as a promising and previously understudied avenue for understanding thyroid cancer progression. However, given the observational nature of this study, limitations related to dataset integration, and lack of multivariable analysis, these results should be interpreted with caution. Further experimental and prospective clinical studies are required to establish the functional significance and potential clinical applicability of *TRMT9B* as a biomarker in thyroid cancer.

## Data Availability

The data discussed in this publication have been deposited in NCBI's Gene Expression Omnibus and are accessible through GEO Series accession numbers GSE6004, GSE3678, GSE299988, GSE104006, GSE50901, and GSE27155.

## References

[1] Endres L, Fasullo M, Rose R (2019). tRNA modification and cancer: potential for therapeutic prevention and intervention. Future Med Chem.

[2] Towns WL, Begley TJ (2012). Transfer RNA methytransferases and their corresponding modifications in budding yeast and humans: activities, predications, and potential roles in human health. DNA Cell Biol.

[3] Svensson I, Björk G, Björk W, Johansson KE, Johansson A (1968). Evidence for enzymatic methylation in vitro of the ribose moiety of RNA. Biochem Biophys Res Commun.

[4] Begley U, Dyavaiah M, Patil A, Rooney JP, DiRenzo D, Young CM (2007). Trm9-catalyzed tRNA modifications link translation to the DNA damage response. Mol Cell.

[5] Begley U, Sosa MS, Avivar-Valderas A, Patil A, Endres L, Estrada Y (2013). A human tRNA methyltransferase 9-like protein prevents tumour growth by regulating LIN9 and HIF1-α. EMBO Mol Med.

[6] Chen HM, Wang J, Zhang YF, Gao YH (2017). Ovarian cancer proliferation and apoptosis are regulated by human transfer RNA methyltransferase 9-likevia LIN9. Oncol Lett.

[7] Endres L, Begley U, Clark R, Gu C, Dziergowska A, Małkiewicz A (2015). Alkbh8 Regulates Selenocysteine-Protein Expression to Protect against Reactive Oxygen Species Damage. PLoS One.

[8] Gu C, Ramos J, Begley U, Dedon PC, Fu D, Begley TJ (2018). Phosphorylation of human TRM9L integrates multiple stress-signaling pathways for tumor growth suppression. Sci Adv.

[9] Wang S, Liu X, Huang J, Zhang Y, Sang C, Li T (2018). Expression of KIAA1456 in lung cancer tissue and its effects on proliferation, migration and invasion of lung cancer cells. Oncol Lett.

[10] Boucai L, Zafereo M, Cabanillas ME (2024). Thyroid cancer: a review. JAMA.

[11] Haugen BR, Alexander EK, Bible KC, Doherty GM, Mandel SJ, Nikiforov YE (2016). 2015 American thyroid association management guidelines for adult patients with thyroid nodules and differentiated thyroid cancer. Thyroid.

[12] Fagin JA, Wells SA (2016). Biologic and clinical perspectives on thyroid cancer. N Engl J Med.

[13] Carnazza M, Quaranto D, DeSouza N, Moscatello AL, Garber D, Hemmerdinger S (2025). The current understanding of the molecular pathogenesis of papillary thyroid cancer. Int J Mol Sci.

[14] Forma A, Kłodnicka K, Pająk W, Flieger J, Teresińska B, Januszewski J (2025). Thyroid cancer: epidemiology, classification, risk factors, diagnostic and prognostic markers, and current treatment strategies. Int J Mol Sci.

[15] Lam AK (2022). Papillary thyroid carcinoma: current position in epidemiology, genomics, and classification. Methods Mol Biol.

[16] Sung H, Ferlay J, Siegel RL, Laversanne M, Soerjomataram I, Jemal A (2021). Global Cancer Statistics 2020: GLOBOCAN Estimates of Incidence and Mortality Worldwide for 36 Cancers in 185 Countries. CA Cancer J Clin.

[17] Nilsson M, Williams D (2016). On the Origin of Cells and Derivation of Thyroid Cancer: C Cell Story Revisited. Eur Thyroid J.

[18] Chen DW, Lang BH, McLeod DS, Newbold K, Haymart MR (2023). Thyroid cancer. Lancet.

[19] Boucai L, Zafereo M, Cabanillas ME (2024). Thyroid Cancer: A Review. JAMA.

[20] Colaprico A, Silva TC, Olsen C, Garofano L, Cava C, Garolini D (2016). TCGAbiolinks: an R/Bioconductor package for integrative analysis of TCGA data. Nucleic Acids Res.

[21] Uhlen M, Zhang C, Lee S, Sjöstedt E, Fagerberg L, Bidkhori G (2017). A pathology atlas of the human cancer transcriptome. Science.

[22] Yuan M, Zhang C, Von Feilitzen K, Zwahlen M, Shi M, Li X (2025). The Human Pathology Atlas for deciphering the prognostic features of human cancers. EBioMedicine.

[23] Pontén F, Gry M, Fagerberg L, Lundberg E, Asplund A, Berglund L (2009). A global view of protein expression in human cells, tissues, and organs. Mol Syst Biol.

[24] Baloch ZW, LiVolsi VA (2018). Special types of thyroid carcinoma. Histopathology.

[25] Ringel MD, Sosa JA, Baloch Z, Bischoff L, Bloom G, Brent GA (2025). 2025 American Thyroid Association Management Guidelines for Adult Patients with Differentiated Thyroid Cancer. Thyroid.

[26] Uhlén M, Fagerberg L, Hallström BM, Lindskog C, Oksvold P, Mardinoglu A (2015). Proteomics. Tissue-based map of the human proteome. Science.

[27] Barrett T, Wilhite SE, Ledoux P, Evangelista C, Kim IF, Tomashevsky M (2013). NCBI GEO: archive for functional genomics data sets—update. Nucleic Acids Res.

[28] Soneson C, Love MI, Robinson MD (2015). Differential analyses for RNA-seq: transcript-level estimates improve gene-level inferences. F1000 Res.

[29] Clough E, Barrett T, Wilhite SE, Ledoux P, Evangelista C, Kim IF (2024). NCBI GEO: archive for gene expression and epigenomics data sets: 23-year update. Nucleic Acids Res.

[30] Vasko V, Espinosa AV, Scouten W, He H, Auer H, Liyanarachchi S (2007). Gene expression and functional evidence of epithelial-to-mesenchymal transition in papillary thyroid carcinoma invasion. Proc Natl Acad Sci USA.

[31] Reyes I, Reyes N, Suriano R, Iacob C, Suslina N, Policastro A (2019). Gene expression profiling identifies potential molecular markers of papillary thyroid carcinoma. Cancer Biomark.

[32] Mohamad Pakarulrazy NF, Abu N, Abdullah Suhaimi SN, Paisol NH, Md Zin RR, MdLatar NH (2025). Gene Expression Analysis of Papillary Thyroid Carcinoma With Lymph Node Metastasis and Radioiodine Refractivity. Cureus.

[33] Minna E, Brich S, Todoerti K, Pilotti S, Collini P, Bonaldi E (2020). Cancer Associated Fibroblasts and Senescent Thyroid Cells in the Invasive Front of Thyroid Carcinoma. Cancers (Basel).

[34] Barros-Filho MC, Marchi FA, Pinto CA, Rogatto SR, Kowalski LP (2015). High Diagnostic Accuracy Based on CLDN10, HMGA2, and LAMB3 Transcripts in Papillary Thyroid Carcinoma. J Clin Endocrinol Metab.

[35] Giordano TJ, Kuick R, Thomas DG, Misek DE, Vinco M, Sanders D (2005). Molecular classification of papillary thyroid carcinoma: distinct BRAF, RAS, and RET/PTC mutation-specific gene expression profiles discovered by DNA microarray analysis. Oncogene.

[36] Chen JH, Faquin WC, Lloyd RV, Nosé V (2011). Clinicopathological and molecular characterization of nine cases of columnar cell variant of papillary thyroid carcinoma. Mod Pathol.

[37] Rossi ED, Faquin WC, Pantanowitz L (2019). Cytologic features of aggressive variants of follicular-derived thyroid carcinoma. Cancer Cytopathol.

[38] Gordon AJ, Dublin JC, Patel E, Papazian M, Chow MS, Persky MJ (2022). American thyroid association guidelines and national trends in management of papillary thyroid carcinoma. JAMA Otolaryngol Head Neck Surg.

[39] Coca-Pelaz A, Shah JP, Hernandez-Prera JC, Ghossein RA, Rodrigo JP, Hartl DM (2020). Papillary Thyroid Cancer-Aggressive Variants and Impact on Management: A Narrative Review. Adv Ther.

[40] Gaertner EM, Davidson M, Wenig BM (1995). The columnar cell variant of thyroid papillary carcinoma. Case report and discussion of an unusually aggressive thyroid papillary carcinoma. Am J Surg Pathol.

[41] Frates MC, Benson CB, Doubilet PM, Kunreuther E, Contreras M, Cibas ES (2006). Prevalence and distribution of carcinoma in patients with solitary and multiple thyroid nodules on sonography. J Clin Endocrinol Metab.

[42] Morganti S, Ceda GP, Saccani M, Milli B, Ugolotti D, Prampolini R (2005). Thyroid disease in the elderly: sex-related differences in clinical expression. J Endocrinol Invest.

[43] Jonklaas J, Nogueras-Gonzalez G, Munsell M, Litofsky D, Ain KB, Bigos ST, Brierley JD, Cooper DS, Haugen BR, Ladenson PW, Magner J, Robbins J, Ross DS, Skarulis MC, Steward DL, Maxon HR, Sherman SI, National Thyroid Cancer Treatment Cooperative Study Group (2012). The impact of age and gender on papillary thyroid cancer survival. J Clin Endocrinol Metab.

[44] Kitana FI, Shobab L (2025). The Interaction Between Sex and Thyroid Cancer Development and Management. Endocrinol Metab Clin North Am.

